# Unintended Consequences of Obesity Pharmacotherapy: A Nutritional Approach to Ensuring Better Patient Outcomes

**DOI:** 10.3390/nu17111934

**Published:** 2025-06-05

**Authors:** Werd Al-Najim, António Raposo, Mona N. BinMowyna, Carel W. le Roux

**Affiliations:** 1 Diabetes Complications Research Centre, School of Medicine, Conway Institute, University College Dublin, D04 V1W8 Dublin, Ireland; carel.leroux@ucd.ie; 2 CBIOS (Research Center for Biosciences and Health Technologies), Universidade Lusófona de Humanidades e Tecnologias, Campo Grande 376, 1749-024 Lisboa, Portugal; 3 College of Education, Shaqra University, Shaqra 11911, Saudi Arabia; m.mwena@su.edu.sa

**Keywords:** energy restriction, micronutrient deficiency, nutritional complications, nutrition support, obesity pharmacotherapy

## Abstract

**Background/Objectives:** Obesity pharmacotherapy vastly improved the treatment of the disease of obesity. However, GLP-1 receptor agonists and GIP/GLP-1 dual agonists may lead to nutritional complications, including severe caloric restriction, micronutrient deficiencies, lean body mass loss, dehydration, and ketosis. We examine these risks and outlines dietitian-led strategies to support improved safety and effectiveness. **Methods:** This narrative review was conducted in three stages: literature search, screening of abstracts and full texts, and synthesis of findings. Searches were carried out in April and May 2025 across PubMed, Embase, Scopus, ScienceDirect, Web of Science, and Google Scholar using keywords related to obesity pharmacotherapy and nutrition. **Results:** Clinical observations and trial data suggest that some individuals may consume fewer than 800 kcal/day during the initial stages of treatment. Prolonged energy and protein deficits can increase the risk of sarcopenia, metabolic dysfunction, and reduce treatment adherence. Additional risks include inadequate micronutrient intake due to reduced dietary variety, dehydration linked to gastrointestinal symptoms and hypodipsia, and rare but serious cases of ketoacidosis. Patients at heightened risk include older adults, those with low baseline muscle mass, and individuals with restrictive eating patterns. **Conclusions**: Obesity medications introduce unique nutritional risks that are not yet addressed by standardised clinical protocols. Registered dietitians play a critical role in assessing intake patterns, monitoring for red flags, and delivering targeted nutritional support. Integrating structured dietary assessment tools, checklists, and risk-specific guidance into pharmacotherapy pathways can enhance safety, promote adherence, and improve long-term outcomes.

## 1. Introduction

Obesity is a chronic, relapsing disease that has reached epidemic proportions globally, affecting over 107.7 million children and 603.7 million adults [1]. Excess adiposity is a major risk factor for a wide range of chronic conditions, including cardiovascular diseases [2,3], type 2 diabetes [2], chronic kidney disease [2], several types of cancer [4], and musculoskeletal disorders [5,6].

Today, multiple effective treatment options are available to manage the disease of obesity, including medical nutrition therapy [7], pharmacotherapy [8], and bariatric surgery [9]. International clinical guidelines now recommend a patient-centered, multi-modal approach, ensuring that people with the disease of obesity are offered the full range of evidence-based interventions according to clinical eligibility, preferences, and response [10].

Among these, bariatric surgery has well-defined nutritional protocols, guiding dietary management before and after surgery to optimize outcomes and prevent nutritional deficiencies [11]. However, similar structured protocols are lacking for obesity pharmacotherapy, despite its growing adoption and promising outcomes. Lessons from bariatric surgery can thus be very instructive [12]. The absence of standardized dietary guidance for pharmacotherapy presents a knowledge gap, particularly as the use of obesity medications continues to rise and brings with it unintended nutritional consequences that may compromise treatment safety.

Glucagon-like peptide-1 receptor agonists (GLP-1 RAs), such as liraglutide and semaglutide, are effective obesity treatments. Semaglutide 2.4 mg, for example, has been associated with mean weight loss exceeding 15% of initial body weight over 68 weeks [13], while liraglutide 3.0 mg typically results in 5–7% weight loss [14]. More recently, tirzepatide, a dual glucose dependent insulinotropic polypeptide (GIP) and GLP 1 receptor agonist, has shown even greater effectiveness, with 22.5% weight loss in adults with obesity [15].

Between 2019 and 2023, there has been a 700% increase in GLP-1 RA prescriptions among U.S. adults without diabetes. This surge reflects growing clinical and consumer demand, driven by both regulatory approvals and off-label prescribing of agents originally indicated for type 2 diabetes. In some cases, these pharmacotherapies have demonstrated weight loss outcomes comparable to bariatric surgery [16].

However, their mechanism of action, which includes delayed gastric emptying and reduction in appetite, can also give rise to poor nutritional intake. Common adverse effects include nausea, vomiting, diarrhea, constipation, early satiety, dyspepsia, hypodipsia, and anorexia [17,18]. While published data on actual caloric intake is limited, one narrative review reported energy intake reductions of up to 39% in individuals receiving GLP-1 receptor agonists compared to placebo [19]. In a recent trial, tirzepatide significantly reduced daily energy intake by approximately 850 kcal/day compared to placebo, primarily through appetite suppression, while also increasing fat oxidation without inducing metabolic adaptation [20]. In clinical practice, some individuals may experience profound appetite suppression, with caloric intake occasionally falling below 800 kcal/day. Such reductions may lead to micronutrient deficiencies and loss of lean body mass, potentially impairing functional capacity and increasing the likelihood of treatment discontinuation due to poor tolerability.

Given these challenges, healthcare professionals, and particularly dietitians, have a critical role in supporting other healthcare professionals and patients. There is an urgent need to develop monitoring frameworks and clinical tools to detect, prevent, and manage nutrition-related side effects, which can be used in primary and secondary care centers. This review aims to examine the nutritional implications of obesity pharmacotherapy and to propose evidence-informed strategies to support patient safety and optimize treatment outcomes.

## 2. Methods

This narrative review was conducted in three stages: performing the literature search, screening abstracts and full texts, and synthesizing the findings. A comprehensive search was carried out in April and May 2025 using the PubMed, Embase, Scopus, ScienceDirect, Web of Science, and Google Scholar databases to identify relevant literature. Only English-language publications were included.

Keywords used in the search included “GLP-1 receptor agonists”, “GIP/GLP-1 dual agonists”, “anti-obesity medications”, “obesity pharmacotherapy”, “nutrition”, “energy restriction”, “micronutrient deficiency”, “sarcopenia”, “ketoacidosis”, and “dietitian”. These terms were combined using Boolean operators (AND and OR) to ensure a comprehensive yet focused search strategy.

After removing duplicate records, abstracts of the remaining articles were screened for relevance to the topic. Full texts were then reviewed to determine eligibility. Studies were included if they reported on nutritional complications, dietary intake, or clinical nutrition management in the context of pharmacological treatment for obesity.

Eligible sources included clinical trials, observational studies, case reports, practice guidelines, and narrative reviews. Relevant findings were summarized and synthesized to support the aims of this review. Particular attention was given to evidence describing reduced caloric intake, nutrient inadequacy, lean mass changes, hydration status, and the clinical role of dietitians both as clinicians but also educators to other healthcare professionals. As this was a narrative review, detailed documentation of the search protocol was not required, and a formal PRISMA flow diagram was not applicable. However, a structured and transparent screening process was followed to minimize selection bias.

## 3. Unintended Health Consequences

**Figure 1:**
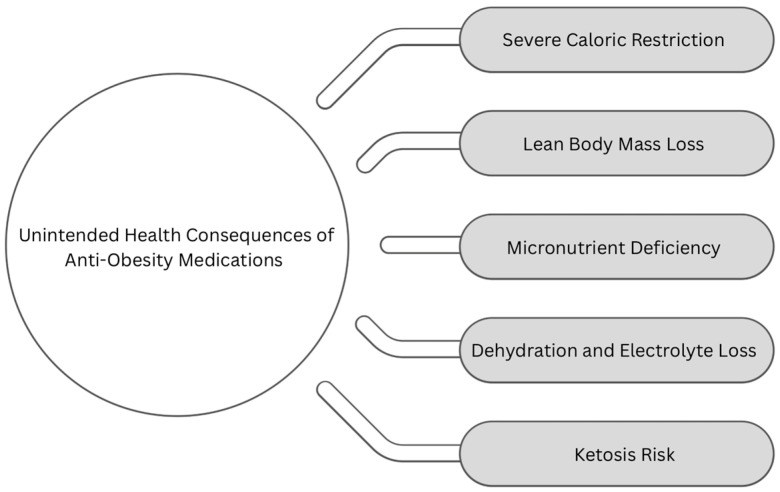
Unintended health consequences of obesity pharmacotherapy.

Nutritional side effects affect a small number of patients after obesity pharmacotherapy, but because the absolute number of patients being treated are increasing, more nutritional side effects are observed in real-world settings. While these medications are effective in inducing weight loss, some patients experience unintended physiological consequences that require targeted nutritional support. This section outlines key health concerns associated with pharmacotherapy, including severe caloric restriction, lean body mass loss, micronutrient deficiencies, dehydration, and ketoacidosis (Figure 1), and presents evidence-informed strategies to support their nutritional management in routine practice.

### 3.1. Severe Energy Deficit and Inadequate Caloric Intake

Obesity medications reduce energy intake, but can also delay gastric emptying, which, in turn, can amplify enhanced satiety [21]. Reduction in appetite can be significant, particularly during the initial titration phase of glucagon-like peptide-1 receptor agonists (GLP-1 RAs) and dual GIP/GLP-1 receptor agonists [21,22]. While reduced caloric intake is a desired therapeutic effect, in a smaller number of individuals, it may become excessive and clinically concerning.

Clinical observations and patient reports suggest that some individuals treated with GLP-1 RAs or dual GIP/GLP-1 agonists may unintentionally consume fewer than 800 kcal per day, especially during the early treatment phase if the patients also experience pronounced gastrointestinal side. While structured very low-calorie diets (VLCDs) exist as medically supervised interventions [23], the spontaneous dietary restriction observed in patients treated with obesity medicines is typically unbalanced in macro- and micronutrient composition. This raises concerns about the risk of malnutrition, fatigue, and metabolic complications in these smaller number of patients who are not being nutritionally monitored.

In clinical trials, gastrointestinal adverse events, including nausea and early satiety, have been reported in up to 74% of participants, with approximately 7–10% discontinuing treatment due to intolerable symptoms [24]. Although actual caloric intake is not routinely reported, these findings underscore the potential for undernutrition in clinical practice.

Patients at higher risk include older adults [25] and individuals with restrictive food beliefs or avoidant/restrictive food intake disorder (ARFID) [26], where substantial reductions in appetite may exacerbate inadequate intake.

### 3.2. Lean Body Mass Loss and Risk of Sarcopenia

Weight loss, particularly when rapid or unaccompanied by adequate protein intake or resistance exercise, is frequently associated with a reduction in both fat mass and lean body mass. While the primary goal of obesity treatment is to reduce excess adiposity, unintended loss of muscle mass can impair resting metabolic rate, compromise physical function, and increase the risk of sarcopenia, especially in older adults or individuals with lower baseline muscle reserves [27,28]. The general principle that each kilogram of weight loss comprises approximately 75% fat and 25% lean mass is widely cited [29], though the actual proportion may vary depending on factors such as age, protein intake, and physical activity.

Several clinical trials of GLP-1 receptor agonists and related pharmacotherapies have reported that a proportion of total weight loss occurs as lean mass, though the magnitude varies by drug, population, and methodology. In the STEP 1 trial, semaglutide treatment resulted in an average lean mass reduction of 6.9 kg (−13.2%) out of a total 15.3 kg (−14.9%) weight loss, representing approximately 45% of the total weight lost as lean mass [13]. In the SURMOUNT-1 trial of tirzepatide, 25.7% of weight lost was attributed to lean mass [15]. Similarly, in the SUSTAIN-8 sub study, patients treated with semaglutide lost 2.3 kg (−4.5%) of lean mass out of a total 5.3 kg (−6.0%) weight loss, equating to 43.4%, although lean mass as a proportion of total body mass increased slightly [30]. These findings are consistent with the widely referenced “quarter FFM rule”, which posits that approximately 25% of weight loss will be fat-free mass (FFM) and 75% will be fat mass [29]. Furthermore, this rule is increasingly viewed as a generalization, as the actual ratio is influenced by factors such as dietary protein intake, degree of caloric restriction, baseline adiposity, physical activity, and individual metabolic state [29]. Moreover, lean mass measurements include not only skeletal muscle but also organ tissue, fluid, and fat-free components of adipose tissue, making it difficult to isolate true muscle loss [31].

The risk of lean mass depletion is further amplified in patients who experience appetite suppression, nausea, or food aversion during treatment, which can lead to reduced overall protein and energy intake [32]. This is of particular concern in older adults, where muscle loss contributes to frailty, impaired glucose regulation, increased fall risk, and decreased quality of life [33]. In addition to the loss of muscle, unintentional and rapid weight loss has been shown to adversely affect bone metabolism, increasing bone turnover and potentially accelerating the onset of osteopenia and osteoporosis, especially in postmenopausal women and older adults [34,35].

Furthermore, individuals with obesity who have undergone multiple weight loss attempts may be particularly vulnerable to long-term shifts in body composition [36]. Each cycle of weight loss often results in some degree of lean mass loss, while subsequent weight regain tends to favor fat accumulation. Over time, this pattern can lead to an unfavorable fat-to-lean mass ratio and may contribute to the development of sarcopenic obesity, a condition associated with reduced strength, metabolic inflexibility, and increased cardiometabolic risk [37]. This underscores the importance of preserving lean mass not only during treatment but also during long-term weight maintenance.

Patients at greater risk include those over age 65, individuals with low baseline muscle mass or physical inactivity, and those with inadequate dietary protein intake. In these populations, lean mass loss may undermine the metabolic and functional benefits of weight reduction unless proactively addressed.

While weight loss induced by GLP-1 receptor agonists often includes reductions in both fat mass and lean body mass, recent clinical trials have demonstrated that these changes do not necessarily compromise functional capacity. In the STEP-HFpEF trial, semaglutide treatment in patients with obesity-related HFpEF led to significant improvements in the 6-min walk distance (6MWD) and Kansas City Cardiomyopathy Questionnaire Clinical Summary Score (KCCQ-CSS), despite concurrent losses in lean mass [38]. Similarly, the SUMMIT trial evaluating tirzepatide reported enhanced exercise tolerance and quality of life measures, even with reductions in lean body mass [39]. These findings suggest that muscle function may be preserved or even improved, despite decreases in muscle mass. Nonetheless, proactive strategies to maintain or enhance muscle mass, such as resistance training and adequate protein intake, may further amplify the functional benefits conferred by these pharmacotherapies [38].

### 3.3. Micronutrient Deficiencies

Emerging evidence suggests that individuals using GLP-1RAs may be at risk of micronutrient inadequacies due to reduced food volume and suboptimal food group intake. Reduction in appetite and gastrointestinal side effects such as nausea and early satiety may contribute to low consumption of fruits, vegetables, whole grains, and dairy, key sources of vitamins and minerals. A recent exploratory study found that participants commonly failed to meet dietary reference intakes (DRIs) for calcium, magnesium, potassium, vitamin D, iron, fiber, and choline, while overconsuming saturated fat and sodium [40].

Although many participants reported making healthier food choices, objective intake data revealed substantial gaps. For example, average fiber intake was 14.5 g/day (nearly half the recommended 28 g/day), and vitamin D intake was just 4 mcg/day. Protein intake fell within the acceptable macronutrient distribution range (AMDR), yet only a minority of participants met the 1–1.4 g/kg/day threshold advised during energy restriction to support lean mass preservation [36]. These trends are likely to persist in the absence of targeted nutritional support.

This is particularly concerning given that people with obesity frequently present with pre-existing micronutrient deficiencies. In a Chilean study of women with severe obesity, 46% were vitamin D deficient, while 13% had blood levels of calcium, iron, and vitamin B12 consistent with deficiency [41]. A Spanish cohort reported that 10% of women were deficient in vitamin B12, 25% in folate, 26% in vitamin D, 68% in copper, and 74% in zinc [42]. Additionally, a separate analysis reported significantly higher rates of anemia, magnesium deficiency, and low vitamin B6 levels in individuals with obesity [43].

Although these studies were conducted among patients undergoing bariatric surgery, the findings likely reflect broader trends in the population with severe obesity. This suggests that individuals commencing GLP-1RA treatment may already have compromised nutrient reserves, placing them at heightened risk of deficiency as overall food intake declines. The usual duration of the substantial decline in calorie intake may be 3–6 months and most people may be able to compensate because of pre-intervention micronutrient stores, but identifying those patients who may be vulnerable pre-intervention is important.

### 3.4. Risk of Dehydration and Electrolyte Imbalance

Dehydration is an under recognized yet clinically significant risk during GLP-1 receptor agonist (GLP-1RA) treatment. Reduction in appetite, gastrointestinal side effects, and reduced fluid intake contribute collectively to this concern. Nausea, vomiting, diarrhea, and early satiety, commonly reported during dose titration, can substantially limit oral fluid intake and lead to fluid loss. In rare cases, this may result in clinically meaningful dehydration or electrolyte disturbances such as hyponatremia or hypokalemia [44].

In a pharmacovigilance analysis comparing dulaglutide, liraglutide, semaglutide, and tirzepatide, it was semaglutide that was associated with the highest risk of fluid reduction-related adverse events, indicating a potential class effect with varying degrees of severity [44]. Preclinical evidence also suggests that GLP-1RAs may directly cause hypodipsia via central nervous system pathways. Animal studies have shown that both endogenous and exogenous GLP-1 reduce fluid consumption by activating CNS GLP-1 receptors, independently of food-related cues [45,46]. These mechanistic findings are supported by clinical observations and real-world case reports of fluid depletion associated with GLP-1Ras [47,48].

Importantly, most studies have focused on doses typically used in type 2 diabetes. There is limited evidence regarding the effects of higher GLP-1RA doses used for obesity, where gastrointestinal symptoms may be more pronounced. This raises questions about whether fluid intake suppression may be more severe at obesity-specific dosing levels.

Reduced thirst cues, often coinciding with appetite suppression, can further diminish hydration behaviors. In clinical practice, patients frequently report sipping only small amounts of water or skipping fluids to avoid worsening nausea or bloating. These patterns, combined with persistent GI symptoms, place individuals at heightened risk of dehydration, particularly older adults or those taking diuretics, antihypertensives, or nephrotoxic medications.

Insensible water loss, the amount of water lost daily through evaporation from the skin and respiratory tract, which is not easily measurable, may also be elevated during obesity treatment. Increased fat oxidation, as observed in patients treated with tirzepatide [20], may require greater metabolic water utilization. The oxidation of triglycerides involves multiple metabolic steps where water is both consumed and produced, contributing to the body’s overall water balance. This added physiological burden may further exacerbate the risk of dehydration, particularly when fluid intake is already reduced due to gastrointestinal symptoms or appetite suppression.

### 3.5. Ketosis and Ketoacidosis Risk

While mild nutritional ketosis can occur during energy restriction and is generally well tolerated, more severe ketone accumulation, including diabetic ketoacidosis (DKA) and euglycemic DKA, has been reported in rare cases among individuals using GLP-1Ras [44]. Appetite suppression delayed gastric emptying, and significant reductions in caloric and carbohydrate intake may contribute to increased ketogenesis, particularly in individuals with diabetes or those taking additional medications that affect insulin action or glucose metabolism [44]. Recent pharmacovigilance data indicate a disproportionate number of adverse event reports involving ketoacidosis across several GLP-1RAs, including semaglutide, liraglutide, dulaglutide, and tirzepatide. Notably, nearly all reported cases were classified as severe in outcome, even when SGLT2 inhibitors or other confounding agents were not involved [44].

A published case report described DKA in a patient with type 1 diabetes mellitus following the use of semaglutide (Wegovy) 1.7 mg. In this case, appetite suppression, along with GLP-1RA-induced inhibition of gluconeogenesis and glycogenolysis, was thought to have contributed to reduced insulin administration and the subsequent development of ketoacidosis [44]. These findings are consistent with a 2023 meta-analysis showing that liraglutide was associated with a significantly increased odds of ketosis in patients with type 1 diabetes (OR 1.8; 95% CI, 1.1–2.8) [49]. Additional analyses have also reported modestly elevated rates of ketoacidosis in GLP-1RA users without concurrent insulin therapy, suggesting a possible class effect that warrants further study [50].

While these events remain rare, the risk may be heightened in individuals receiving higher doses of GLP-1RAs for obesity treatment, especially when combined with very low carbohydrate intake, prolonged vomiting, or inadequate insulin dosing. Patients with type 1 diabetes, insulin deficiency, or concurrent SGLT2 inhibitor use are particularly vulnerable.

Risk Factors for Ketosis-Related Complications

▪ Type 1 diabetes or late-stage type 2 diabetes with reduced insulin reserve;
▪ Caloric intake less than 800 kilocalories per day with insufficient carbohydrate intake;
▪ Concomitant SGLT2 inhibitor therapy;
▪ Prolonged vomiting, dehydration, or missed insulin doses;
▪ Use of high-dose GLP-1RAs for obesity outside of diabetes indications.

## 4. Nutritional Management and Education for Healthcare Professionals

Effective nutritional management during obesity pharmacotherapy is critical to ensure safe and sustainable weight loss, preserve lean body mass, and prevent micronutrient deficiencies or metabolic complications. As reduction in appetite and gastrointestinal symptoms can reduce both the volume and variety of food consumed, proactive monitoring by registered dietitians or healthcare professionals with adequate training is essential.

Clinical nutrition support should priorities the following goals:

1. Maintain minimum energy and protein intake to prevent undernutrition and preserve muscle mass.
2. Promote nutrient-dense eating patterns aligned with national dietary guidelines (e.g., MyPlate or the Food Pyramid).
3. Prevent dehydration and electrolyte imbalance through regular fluid intake guidance.
4. Monitor carbohydrate intake to avoid ketosis-related complications, especially in individuals with diabetes.
5. Identify early signs of nutritional risk, including restrictive eating behaviors, gastrointestinal intolerance, or fatigue.

In addition to clinical monitoring, it is essential that patients are informed about the potential nutritional risks associated with obesity medications. Clear communication about expected changes in appetite, potential gastrointestinal side effects, and signs of dehydration or nutritional deficiency can empower patients to engage in self-monitoring and report symptoms early. Incorporating these discussions into routine counselling helps foster shared decision-making, improves adherence, and supports the early identification of complications that may otherwise go unrecognized in primary care settings.

**Figure 2:**
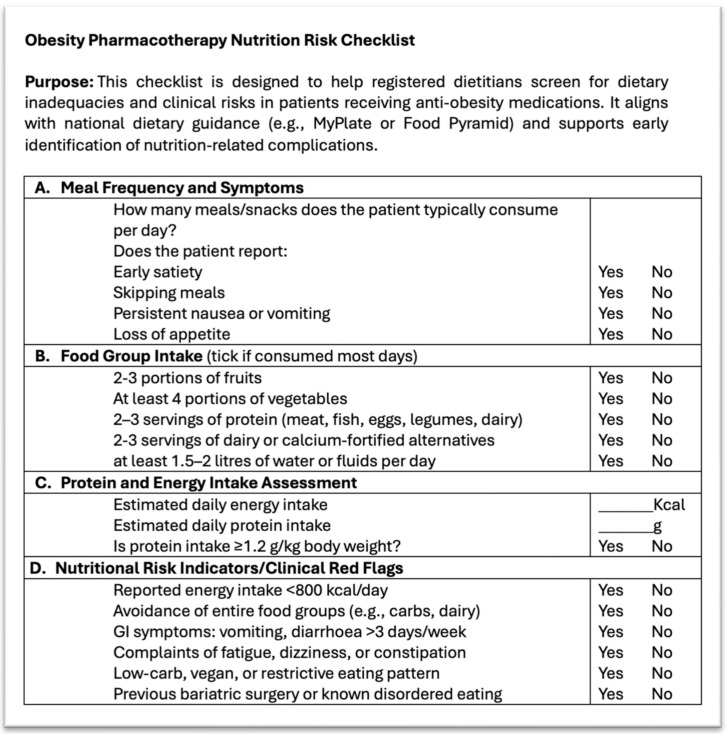
Nutritional risk and intake monitoring checklist for patients on obesity pharmacotherapy.

To support clinical implementation, we developed an original, practice-oriented checklist to assist dietitians and other healthcare professionals in identifying and addressing common nutrition-related risks in individuals receiving obesity medications. This tool was created based on a synthesis of clinical trial findings, case reports, and professional guidelines, and is designed to facilitate routine monitoring and timely intervention. It focuses on five key areas of concern: severe caloric restriction, loss of lean body mass, micronutrient inadequacy, dehydration and electrolyte imbalance, and the risk of ketosis. For each category, it outlines specific nutritional strategies that can be used to guide personalized care. Although this checklist has not yet been formally validated, it provides a structured approach to enhance treatment safety, improve adherence, and support effective dietary counselling in clinical practice (Figure 2).

### Targeted Nutritional Strategies by Risk Area

Building on the checklist introduced in Figure 2, this section presents specific dietary strategies aligned with each identified risk area. These targeted recommendations are summarized in Table 1 and are intended to guide clinical decision-making and personalized nutrition planning during pharmacotherapy for obesity. Table 1 serves as a practical tool to support the early detection and management of complications such as undernutrition, lean mass loss, micronutrient deficiencies, dehydration, and ketosis. Strategies should be adapted based on individual patient needs, treatment stage, symptom profile, and comorbidities.

## 5. Limitations

As a narrative review, this study has several limitations. The literature search was not conducted using a systematic or meta-analytic approach, and no formal quality appraisal tools were applied to assess the risk of bias in individual studies. The inclusion of studies was based on relevance to the topic and clinical judgement, which may introduce subjectivity. Additionally, while this review draws on data from clinical trials, case reports, and expert guidelines, the evidence base for some nutritional risks remains limited and heterogeneous.

The proposed checklist and targeted strategies were developed based on the synthesis of existing literature and expert practice considerations. However, the checklist has not yet undergone validation or empirical testing in clinical settings. As such, its effectiveness in improving patient outcomes has not been formally evaluated. Future research should focus on testing the implementation, acceptability, and clinical utility of structured nutritional tools in populations receiving obesity pharmacotherapy.

## 6. Conclusions

As obesity medications continue to evolve and become more widely adopted, attention must shift from weight loss alone to long-term health gain. While agents such as GLP-1 receptor agonists and dual GIP/GLP-1 agonists offer transformative benefits in terms of weight loss, they can also introduce unintended nutritional consequences that may compromise treatment sustainability.

This review highlights the most common nutrition-related risks associated with obesity medications, ranging from inadequate caloric intake and lean mass loss to micronutrient deficiencies, dehydration, and ketosis. These risks are rare but often under-recognized in clinical settings and may be compounded by pre-existing vulnerabilities among certain people with obesity.

Registered dietitians play a vital role in addressing these challenges. Through routine monitoring, dietary counselling, and early intervention, they can help patients meet nutritional needs, preserve muscle mass, and manage side effects that may otherwise reduce adherence or lead to treatment discontinuation. Practical tools, such as screening checklists and risk-specific guidance, can support the integration of nutrition into pharmacotherapy pathways. Dieticians should also educate other healthcare professionals to allow these medications to be safely used in primary care settings. Equally, patients should be made aware of potential nutritional risks in order to support informed decision-making, encourage self-monitoring, and promote early reporting of symptoms.

To maximize the safety and effectiveness of obesity medications, care models must include structured nutritional support as a core component. Such support should be personalized to account for comorbidities, baseline nutritional status, treatment response, and individual risk profiles. Future research should continue to refine dietary recommendations specific to pharmacotherapy use and evaluate the outcomes of personalized, nutrition-led interventions in this population to allow the benefits of these medications to be realized at scale.

## Figures and Tables

**Table 1:** Targeted nutritional strategies for common risk areas during obesity pharmacotherapy. This table summarizes practical, evidence-informed dietary interventions to support registered dietitians and other healthcare professionals in managing five priority nutritional risks: severe caloric restriction, lean body mass loss, micronutrient deficiency, dehydration and electrolyte imbalance, and ketosis risk.

Risk Area	Recommended Nutritional Strategies
Severe Caloric Restriction	Encourage a minimum intake of ~1200 kcal/day for females and 1500 kcal/day for males; Recommend energy-dense, nutrient-rich foods; Monitor for signs of fatigue, dizziness, or reduced function.
Lean Body Mass Loss	Priorities protein intake of 1–1.4 g/kg/day, spaced across meals/snacks; Encourage resistance exercise; Consider oral nutrition supplements (ONS) if intake is inadequate.
Micronutrient Deficiency	Emphasize high-bioavailability foods (e.g., red meat, dairy, fortified cereals, leafy greens); Recommend multivitamins and targeted supplements based on intake or lab results.
Dehydration and Electrolyte Loss	Promote daily fluid intake of 1.5–2 L; Adjust intake for climate, activity, and comorbidities; Suggest oral rehydration solutions for persistent GI symptoms Monitor for hypotension, especially in those on antihypertensives or diuretics.
Ketosis Risk	Ensure carbohydrate intake meets at least 130 g/day Avoid combining pharmacotherapy with ketogenic or very low-carbohydrate diets unless medically supervised.

## Abbreviations

The following abbreviations are used in this manuscript:

GLP-1 RAs	Glucagon-like peptide-1 receptor agonists,
BMI	Body mass index
GIP	Glucose-dependent insulinotropic polypeptide
VLCD	Very-low-calorie diets
ARFID	Avoidant/restrictive food intake disorder
AMDR	Acceptable macronutrient distribution range
DKA	Diabetic ketoacidosis
euDKA	Euglycemic diabetic ketoacidosis
ONS	Oral nutrition supplements
